# Shifting brucellosis risk in livestock coincides with spreading seroprevalence in elk

**DOI:** 10.1371/journal.pone.0178780

**Published:** 2017-06-13

**Authors:** Angela Brennan, Paul C. Cross, Katie Portacci, Brandon M. Scurlock, William H. Edwards

**Affiliations:** 1 Wyoming Cooperative Fish and Wildlife Research Unit, Department of Zoology and Physiology, University of Wyoming, Laramie, Wyoming, United States of America; 2 U.S. Geological Survey, Northern Rocky Mountain Science Center, Bozeman, Montana, United States of America; 3 U.S. Department of Agriculture, Animal and Plant Health Inspection Service-Centers for Epidemiology and Animal Health, Fort Collins, Colorado, United States of America; 4 Wyoming Game and Fish Department, Pinedale, Wyoming, United States of America; 5 Wyoming Game and Fish Department, Laramie, Wyoming, United States of America; Universidade de Aveiro, PORTUGAL

## Abstract

Tracking and preventing the spillover of disease from wildlife to livestock can be difficult when rare outbreaks occur across large landscapes. In these cases, broad scale ecological studies could help identify risk factors and patterns of risk to inform management and reduce incidence of disease. Between 2002 and 2014, 21 livestock herds in the Greater Yellowstone Area (GYA) were affected by brucellosis, a bacterial disease caused by *Brucella abortus*, while no affected herds were detected between 1990 and 2001. Using a Bayesian analysis, we examined several ecological covariates that may be associated with affected livestock herds across the region. We showed that livestock risk has been increasing over time and expanding outward from the historical nexus of brucellosis in wild elk on Wyoming’s feeding grounds where elk are supplementally fed during the winter. Although elk were the presumed source of cattle infections, occurrences of affected livestock herds were only weakly associated with the density of seropositive elk across the GYA. However, the shift in livestock risk did coincide with recent increases in brucellosis seroprevalence in unfed elk populations. As increasing brucellosis in unfed elk likely stemmed from high levels of the disease in fed elk, disease-related costs of feeding elk have probably been incurred across the entire GYA, rather than solely around the feeding grounds. Our results suggest that focused disease mitigation in areas where seroprevalence in unfed elk is high could reduce the spillover of brucellosis to livestock. We also highlight the need to better understand the epidemiology of spillover events with detailed histories of disease testing, calving, and movement of infected livestock. Finally, we recommend using case-control studies to investigate local factors important to livestock risk.

## Introduction

Brucellosis is an important zoonotic disease of livestock and wildlife that affects more than 500,000 people annually across the globe [1,2]. Though incidence of brucellosis in humans has dramatically declined where the disease has been reduced or eliminated from livestock [1,2], disease control can be cost prohibitive and complicated by disease re-emergence in countries where unregulated animal movement occurs across borders [1–3]. Spillover infections from wildlife reservoirs can also fuel local outbreaks in livestock, ultimately limiting the success of eradication programs. In the United States, for example, an expensive eradication program reduced prevalence of brucellosis in adult cattle from 11.5% in 1934 to a national herd prevalence of 0.0001% in 2007 [4], but complete eradication was not possible due to periodic and recently increasing [5] spillover from wild elk of the Greater Yellowstone Area (GYA; see [5–7] regarding evidence of elk to cattle transmission). This has forced the livestock industry to impose interstate trade restrictions and costly testing requirements [8] to identify infected livestock and reduce the potential for additional outbreaks as GYA livestock are moved to outside markets and grazing areas. Thus, it has become increasingly important to better understand risk to GYA livestock in an effort to refine mitigation efforts and reduce spillover from elk.

Prior to 2005, elk exposure to *Brucella abortus*, the bacteria that causes brucellosis in the GYA, was highest on 23 winter feeding grounds in western Wyoming where elk are supplementally fed to sustain large elk populations and limit comingling with cattle [9]. Roughly 23,000 elk are fed each winter on the feeding grounds [10], and from 1991–2009 22% of fed elk were seropositive for *B*. *abortus*, compared to 3.7% of unfed elk in the brucellosis endemic area within Wyoming [11]. Feeding grounds have been a hotspot for brucellosis because elk densely aggregate on feedlines during a large part of the *B*. *abortus* transmission period. Brucellosis causes abortions in roughly 50% of pregnant female elk the first year post-infection [12], and can then be transmitted to other elk or livestock who ingest or feed near infected aborted materials [13]. Abortions generally occur between mid-February and late-May [14] when elk are on feeding grounds or on lower elevation winter or spring transitional ranges that tend to be closer to livestock properties. Infectious live births are also possible [12] and could extend the transmission period into late June [14], but are thought to be less important for transmission than abortions because environmental conditions in late May and June do not favor the persistence of *B*. *abortus* [15] and because elk typically select concealed sites for parturition [16]. In addition, research using vaginal implant transmitters (VITs) in feed ground elk from 2006–2016 found that 40% (12/30) of expelled VITs attributed to abortion events were culture or PCR positive for *B*. *abortus*, whereas only 4% (4/104) of VITs attributed to parturition events were positive (Wyoming Game and Fish Department [WGFD] *unpublished data*). The duration of exposure to infectious materials in the environment is thought to be short-lived because scavengers tend to remove the items quickly [17]. It is possible for *B*. *abortus* to persist in the environment for weeks to months in shaded, cold, wet environments, however, when the fetal materials are not removed by scavengers [15].

Livestock surrounding elk feeding grounds were commonly thought to be at the greatest risk of contracting brucellosis because of their proximity to fed elk during the transmission period. However, this landscape of risk may be shifting with recent increases in brucellosis seroprevalence in various unfed elk populations distant from the feeding grounds. Over the last decade a number of unfed elk populations have exhibited *B*. *abortus* exposure levels similar or even higher than that of elk attending feeding grounds, and evidence suggests that these high levels of brucellosis seroprevalence are positively related to elk densities and group sizes on native wintering ranges [18–20]. Growing elk populations across portions of the GYA could also increase the likelihood of elk-livestock comingling. As a result, livestock risk may be expanding from feeding grounds to other areas of the GYA where unfed elk densities and seroprevalence are high. Studies of brucellosis in unfed elk, however, have been conducted in only portions of the GYA, thereby limiting our knowledge of the regional spatial and temporal variability of elk exposure to *B*. *abortus* and the effects on spillover to livestock across the region.

Here, we examined the elk characteristics (fed/unfed status, brucellosis seroprevalence, and density of seropositive elk) associated with brucellosis-affected livestock herds (herds having at least one animal test positive for brucellosis) using 25 years of elk seroprevalence data and population trend counts collected across the GYA in Montana, Wyoming, and Idaho. As elk seroprevalence and density have been recorded intermittently, we conducted the analysis using Bayesian multilevel models with Markov Chain Monte Carlo (MCMC) estimation to predict missing and poorly sampled years, assess spatiotemporal variation in elk seroprevalence, and account for uncertainty across all model parameters. We expected seroprevalence and density of seropositive elk to be strongly associated with affected livestock herds, and for these effects to interact with fed/unfed status. We also expected incidents of spillover to livestock to be increasing at a faster rate in areas without feeding grounds. We examined snowpack and forage quality (using Normalized Difference Vegetation Index [NDVI]) because they can influence elk distributions [21–23], timing of spring elk migration [24] and livestock turnout on grazing allotments that often overlap with elk habitat. We expected a negative relationship between snow levels and livestock risk and a positive relationship between NDVI and livestock risk. Additionally, we summarized epidemiological reports for each affected livestock herd to identify shared characteristics among spillover seasons and methods of detection. Commonalities could help managers focus brucellosis mitigation efforts on the riskiest period and areas for livestock and target detection strategies.

## Materials and methods

**Fig 1:**
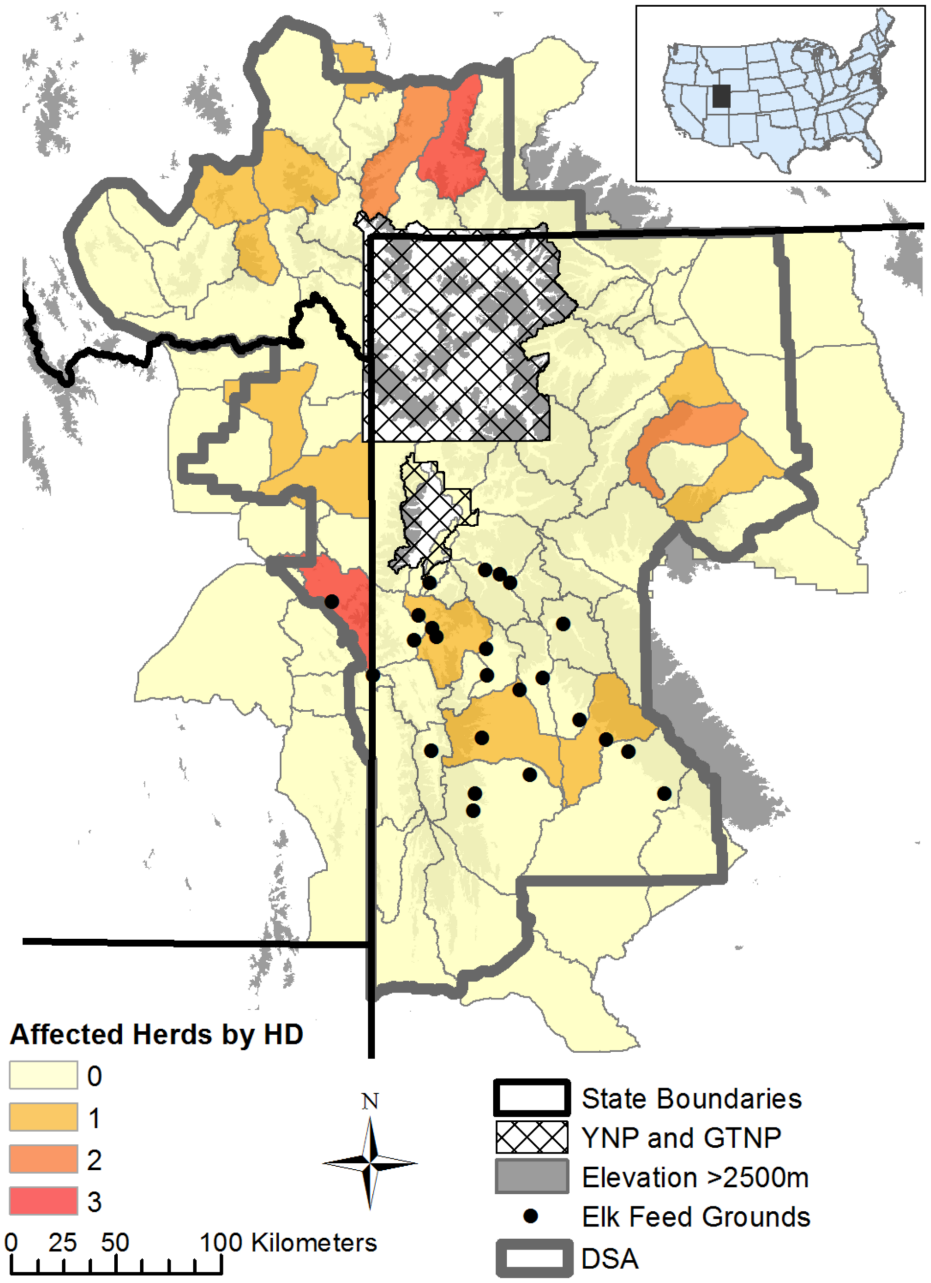
Map of study area and number of brucellosis-affected livestock herds (1990–2014) per hunt district (HD). HDs in the study area overlap with the designated surveillance area (DSA) for brucellosis in livestock. YNP = Yellowstone National Park; GTNP = Grand Teton National Park. Inset: Map of contiguous United States and study area.

We examined occurrences of brucellosis-affected livestock herds (including cattle and domestic bison) during 1990–2014 within the region referred to as the Designated Surveillance Area (DSA), where additional livestock testing and mitigation occurs because of brucellosis spillover risk to livestock [4]. Montana, Idaho and Wyoming each independently design and implement a Designated Surveillance Area (DSA) that is approved by the United States Department of Agriculture (USDA). These DSAs, which we refer to collectively as the DSA, encompass the GYA and most surrounding areas where brucellosis exposure has been detected in wild elk (Fig 1). We obtained epidemiologic reports for each brucellosis-affected cattle or domestic bison herd from the USDA-Animal and Plant Health Inspection Service (USDA-APHIS) to identify the probable spillover season and the detection method that was used to identify infected livestock (see Table A in S1 Appendix for a description of detection methods). Spillover seasons were described as winter (February–March), spring (April–May), or summer (June), but often specific months were not reported.

We obtained locations (identifying main ranch or grazing areas) for each affected livestock herd from USDA-APHIS and assigned these locations to one of 82 elk management units, called hunt districts (HD), that overlapped with the DSA (Fig 1). We used HD as the sampling unit to examine elk factors important to livestock risk to avoid publication of sensitive personal information at finer resolutions, and because the HD is the finest scale that elk ecological data are recorded across all three states. All HDs in our study contained livestock grazing allotments or pastures on public or private land, respectively. Livestock densities were only available at the larger county level (S1 Fig), and so were not included in this analysis.

Brucellosis surveillance in elk has been ongoing for decades via serologic tests that measure an animal’s exposure to *B*. *abortus*. Though seroprevalence cannot distinguish between current or previous infection, it can be useful for tracking increasing trends in brucellosis, as roughly 54% (106/197) of seropositive feed ground elk were culture positive [25]. For our study, the WGFD, Montana Fish, Wildlife and Parks, and Idaho Fish and Game provided serology data from 17,342 hunter-killed and research-captured yearling and older female elk blood samples collected across the DSA from 1990–2014. The state agencies developed serologic profiles for each elk using a battery of diagnostic tests outlined in S2 Appendix. Which tests were used varied slightly by state and year, but in most cases seropositivity was determined from multiple tests run in parallel following the U.S. Department of Agriculture Uniform Methods and Rules for brucellosis in Cervidae [26]. Changes in test accuracy through time have not been examined in elk (see [27–29] for available information regarding *B*. *abortus* serological test sensitivity and specificity in elk), but minor improvements in accuracy are thought to have small effects on the overall spatial and temporal pattern of increasing seroprevalence (S2 Fig). In addition, several tests such as the card and rivanol tests have been used consistently over time, and results from those tests alone support a pattern of increasing seroprevalence (S2 Fig). Therefore, we used the states’ determination of seropositivity for each elk to estimate elk seroprevalence per HD-year in the study.

Data from only females were included in the analysis to avoid confounding effects of sex and to concentrate on the segment of the population with the greatest effect on transmission [12]. We used only the first determination of seropositivity for radio-collared elk that were targeted for testing across multiple years (i.e., non-randomly sampled after the first year; only 30 repeated serologic determinations were not included following this criteria), but for elk that were sampled multiple times and not specifically targeted, we used all serologic determinations in our analysis.

We calculated elk density on the winter range (elk/km^2^) using annual all age and sex trend counts and winter range polygons provided by state wildlife agencies. We use these elk trend counts as indices of female elk densities, because female elk only trend counts were not available, and sex-age classification data were often missing or lacked adequate sample sizes. Trend counts were often collected at the HD level, but in some areas we aggregated counts to the HD level when data were collected at finer scales. In other areas trend counts were recorded in aggregate across multiple HDs (e.g., 500 elk across HDs 1 and 2), and in these cases we divided the aggregate count by number of HDs (e.g., from previous example: 250 elk in HD 1 and 250 elk in HD 2) because there was no information about how to split the data. HDs with no winter range were assumed to have zero wintering elk, though no official counts were collected in these areas to confirm a lack of elk. The methods used to delineate winter range included a combination of biologist opinion, elk locations collected during historic or current winter trend counts, and elk GPS data collected during the winter, but the degree that each method was used to generate winter range polygons varied by state. The extent that winter ranges changed over time was also unknown for most HDs in the study area. We focused on the winter range, however, because it is the best available data that describes the area that elk occupy during the transmission period (excludes highly developed areas and high elevation summer range). Winter range elk density and elk seroprevalence were then used to estimate the density of seropositive elk, as described in the statistical analysis methods below.

We identified the HDs containing feeding grounds, including the 22 state-operated feeding grounds in western Wyoming, the federally operated National Elk Refuge (NER) in Jackson, Wyoming, and a temporarily-operated feeding ground in Idaho (Fig 1) to examine the effects of fed/unfed status on occurrences of brucellosis-affected livestock herds. The Idaho feeding ground stopped operating in 2004–2005, and therefore we did not include it as a feeding ground HD beyond 2005.

To understand snow effects on livestock risk, we used daily snow data recorded from the SNOTEL station nearest to each HD and calculated the average springtime snow water equivalents (SWE; cm) from April through May. Though SNOTEL stations are typically located at elevations higher than elk winter range [30], we use SNOTEL data as an index of winter severity to help us understand regional spatial and temporal variability in snow. We examined average May NDVI at the HD-level using MODIS (Moderate Resolution Imaging Spectroradiometer) 250-m resolution, 16 day composites of NDVI in May (May 15/16 NDVI panels) because this is roughly the period when migration occurs and has been identified as an important month to brucellosis transmission risk [14].

We also conducted an exploratory examination of the association between brucellosis-affected livestock herds and wolf abundance, and report wolf effects that were neither strongly positive nor negative (S3 Appendix). We considered this analysis because wolves may indirectly affect livestock brucellosis through effects on elk distributions and aggregation patterns (e.g., elk form larger groups in open areas with more wolves; [23,31]). At the scale of our study, however, we could not differentiate between wolf effects on elk group size versus elk group size effects on wolf abundance (as aggregating elk can attract wolves), and not understanding the difference could result in misleading inferences about how wolves relate to the spillover of brucellosis from elk to livestock. Elucidating the relationship between wolf abundance and increasing livestock risk could also be complicated by coincident increases in wolf abundance since reintroduction in 1995 and 1996 (i.e., increasing numbers of brucellosis-affected livestock herds may be coincident with but not explained by increasing wolf abundance).

### Statistical analysis

We examined seven different Bayesian hierarchical models with MCMC estimation to evaluate associations with brucellosis-affected livestock herds (models listed in Table 1). We used Bayesian models rather than standard maximum likelihood methods because they allowed us to build hierarchical models and account for uncertainty in all estimated parameters [32]. We used a vector of response variables ***y*** composed of zeros and ones (zero = no *B*. *abortus*-positive livestock herds were detected; one = a *B*. *abortus*-positive livestock herd was detected) to describe the occurrence of livestock brucellosis in sampling unit *j* (HD 1, 2…82) and year *i* (1990–2014). We fit a binomial model to ***y*** to estimate the probability of an affected HD (i.e., elk hunt district [HD] having affected livestock detected) ***p*** and account for uncertainty in the observation process. We then used the following general logistic regression format to estimate covariate effects: logit(***p***) = **X*β* + Zb**, where **X**_[(*i* x *j*) x *k*]_ is a matrix of covariates, ***β*** is a column vector of length *k* regression coefficients, **Z**_[(i x j) x j]_ is a matrix of sampling unit indicators, and **b** is a column vector of length *j* sampling unit specific random effects. We used random effects in the models to account for unmeasured sampling unit effects. We assumed that prior distributions for ***β*** coefficients were normally distributed with mean of zero and precision of 0.0001, and that the prior distribution for **b** was normally distributed with a mean of zero and a standard deviation that was uniformly distributed from 0 to 20. We used these noninformative priors because little was known about the parameters [32].

**Table 1:** Parameter estimates (log odds) for models of brucellosis-affected hunt districts (HD).

Model	Main Effect: Posterior mean (95% CI)	Fed Effect: Posterior mean (95% CI)	Interaction: Posterior mean (95% CI)
Elk Seroprevalence + RE	**0.05** **(0.0004, 0.10)**	-	-
Seropositive Elk Density + RE	0.14 (-0.06, 0.34)	-	-
Year + RE	**0.20** **(0.11, 0.30)**	-	-
NDVI + RE	-0.008 (-0.05, 0.03)	-	-
Elk Seroprevalence × Fed + RE	**0.13** **(0.06, 0.22)**	1.7 (-1.4, 5.0)	**-0.15** **(-0.30, -0.03)**
Year × Fed + RE	**0.27** **(0.14, 0.40)**	3.1 (-1.1, 6.9)	-0.17 (-0.37, 0.05)
Sp. SWE × Fed + RE	-0.10 (-0.39, 0.16)	1.0 (-1.7, 3.4)	-0.67 (-2.0, 0.27)

Notes: Brucellosis-affected HD refers to the probability of an HD having brucellosis-affected livestock detected. Main effects describe the log odds of a brucellosis-affected HD per 1 unit increase in the explanatory variable. Interactions describe the difference in main effect log odds for fed elk compared to unfed elk. Seroprevalence unit: 1 percent. Seropositive elk density unit: 1 elk/km^2^. Sp. SWE (average spring snow water equivalent) unit: 10 cm. NDVI unit: 0.01. Bold items indicate 95% credible intervals did not overlap zero. RE = Random effect. Fed = elk feeding ground present. The spring SWE model dataset was subset to exclude years in which SNOTEL stations were not present (N = 1885). The spring NDVI model dataset was subset to exclude years that MODIS 16 day composite panels were not available (N = 1148). For other models, N = 2050.

**Fig 2:**
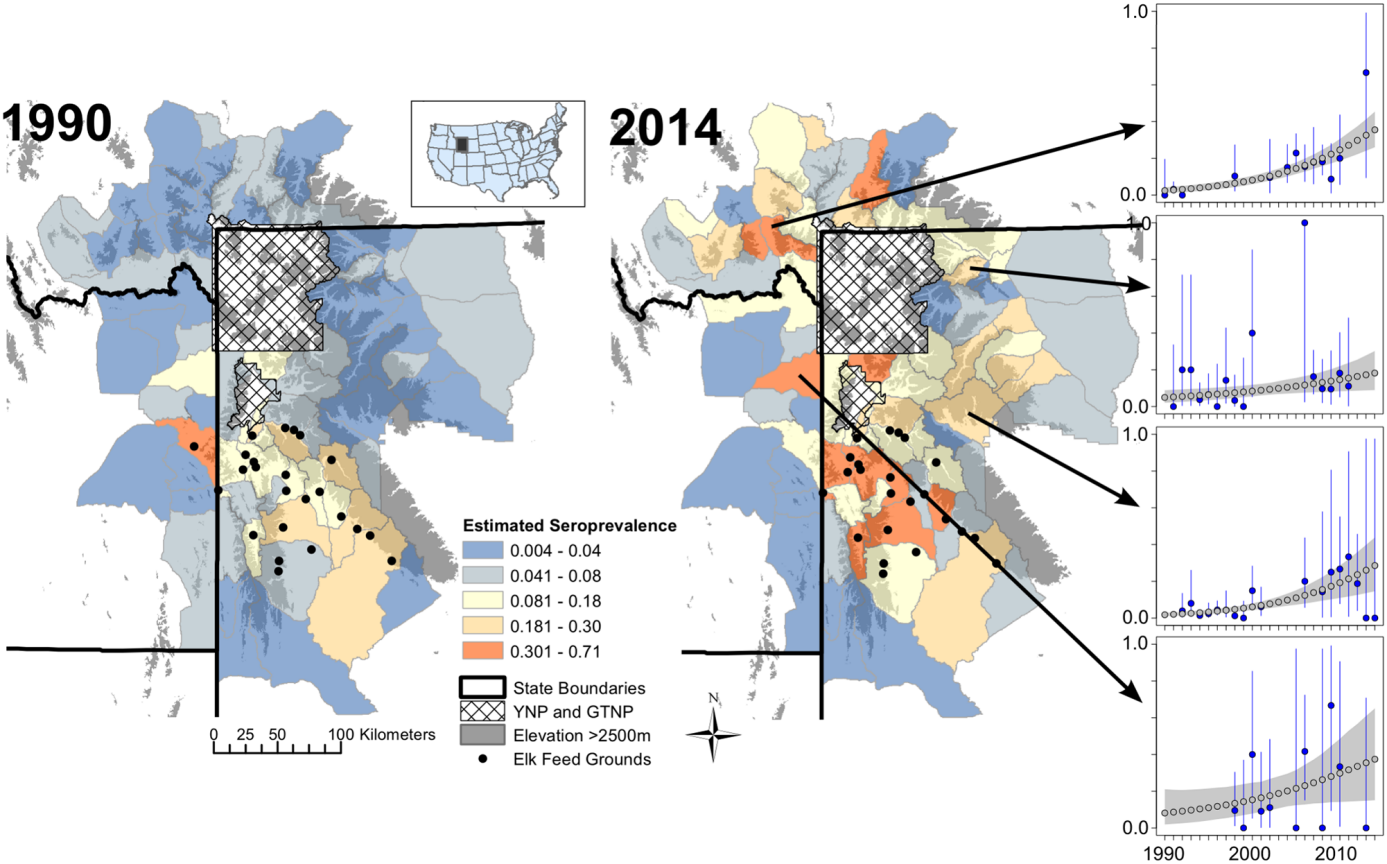
Estimated and empirical brucellosis seroprevalence in elk per elk hunt district (HD). Maps depict estimated brucellosis seroprevalence in elk in 1990 and 2014 for HDs in the study area. YNP = Yellowstone National Park; GTNP = Grand Teton National Park. Inset: Map of contiguous United States and study area. Small panels on the right depict modeled (grey) and empirical (blue) seroprevalence (y axis; range from 0 to 1) over time (x axis; range from 1990 to 2014) from a subset of the study area. Arrows show which elk hunt district corresponds to each panel. Blue lines are 95% binomial confidence intervals, and grey shaded regions are 95% credible intervals.

Out of 2050 HD-years included in the study (82 HDs and 25 years), there were 1283 HD-years missing seroprevalence data and 683 HD-years missing elk counts. There were also years where seroprevalence was based on relatively small sample sizes (e.g., Fig 2). Thus, in models examining the effects of elk seroprevalence on livestock risk, we also modeled seroprevalence as a function of time to estimate missing and poorly sampled years, and examine spatiotemporal variation in elk exposure to *B*. *abortus* across the entire affected region. Previous studies of brucellosis in elk suggest that the temporal dynamics of *B*. *abortus* are slow enough to effectively estimate increases over time despite years of missing data [18,19]. For models examining the effects of seropositive elk density on livestock risk, we incorporated models of elk seroprevalence and elk winter range density as a function of time to estimate missing data and calculate seropositive elk density (seroprevalence × density).

To model seroprevalence, let *s* be the annual number of seropositive elk *i* (for years 1990–2014) in sampling unit *j* (HD 1, 2…82). We modeled *s* as a binomially distributed response variable, where *δ* was the probability of elk testing positive and *n* was the total number of elk that were tested. With the logit link, we estimated *δ* using the following framework: logit(*δ*) = *θ*_0_ + *θ*_1_x_ij_ + *υ*_0j_ + *υ*_1j_x_ij_, where *θ*_0_ was the common intercept, *θ*_1_ was the fixed time effect, x_*ij*_ was year, *υ*_0j_ was a random intercept term by sampling unit, and *υ*_1j_ was a random slope term by sampling unit. Random effects were used to better estimate parameters for HDs with many missing years of data (i.e., to shrink poorly sampled regions toward the global mean; [32]). We also used noninformative prior distributions on all parameters in this model. For *θ*_0_ and *θ*_1_ we assumed that prior distributions were normally distributed with mean of zero and precision of 0.0001, and that prior distributions for *υ*_0j_ and *υ*_1j_ were normally distributed with a mean of zero and a standard deviation that was uniformly distributed from 0 to 20. Using the Bayesian framework, we predicted values of *δ* from the posterior predictive distribution, and then used *δ* as an explanatory variable of affected livestock herds.

To model elk density and predict missing data, let *d* be the square root transformed empirical annual winter range elk density *i* (for years 1990–2014) in sampling unit *j* (HD 1, 2…82). This square root transformation allowed us to model *d* as a normally distributed response variable, with mean *μ* and standard deviation *σ*. We then estimated *μ* from a linear regression model, using the same framework of fixed and mixed effect predictors described above in our model of seroprevalence. Also following the seroprevalence model, we used noninformative prior distributions on all parameters. For fixed effect coefficients we assumed that prior distributions were normally distributed with mean of zero and precision of 0.0001, and that prior distributions for mixed effect coefficients were normally distributed with a mean of zero and a standard deviation that was uniformly distributed from 0 to 20. We predicted values of *μ* from the posterior predictive distribution. We then back-transformed (squared) *μ* and multiplied it by *δ* from the seroprevalence model to generate predicted values of seropositive elk density. We used these predicted values of seropositive elk density as an explanatory variable of affected livestock herds.

All models were fit using the R2WinBUGS package [33] to call WinBUGS version 1.4.3 from R version 3.1.2 [34]. We ran three MCMC chains of 150,000 iterations, discarding the first half of each chain. We assessed convergence from sample trace plots and from calculation of the scale reduction factor ($\hat{\text{R}}$) used to compare within and among chain variance. $\hat{\text{R}}$ values of 1 to 1.1 for all parameters typically indicate convergence [32]. We also conducted posterior predictive checks to assess model fit. We visually compared the posterior predictive distributions of standardized residuals of observed and predicted occurrences of affected HDs (where an even distribution around a 1:1 line indicates the model fit the data) and quantified the proportion of times standardized residuals of predicted data were greater than the standardized residuals of observed data (i.e., Bayesian p-values, where values close to 0.5 indicate good model fit; [35]). We also compared the deviance information criterion (DIC) estimated between the models of interest listed in Table 1 and their corresponding null model (intercept only) to roughly assess whether models with predictors were better supported by the data than null models. We did not compare DIC across models of interest because the response variables were different for different models (i.e., N differed across models; see notes in Table 1 for details).

A standard for epidemiological studies using binary response variables is for at least 10 events of disease per explanatory variable (EPV) to reduce overfitting and coefficient bias [36], though it has been argued that five to nine EPV is an acceptable lower limit [37]. In our study, events were rare (21 occurrences of affected livestock herds) and we used models with 1 or 3 fixed effect variables plus random effects on sampling unit (82 HD units). This addition of random effects substantially affected the number of EPV. Therefore, we compared results from these models to models without random effects, where the number of EPV ranged from 5.25 to 21, in an effort understand coefficient stability after the addition of random effects.

## Results

There were 21 brucellosis-affected livestock herds between 2002 and 2014, nine of which occurred in Montana, seven in Wyoming, and five in Idaho (Fig 1). Only four of the affected livestock herds occurred in elk hunt districts (HDs) with operating feeding grounds (three in Wyoming and one in Idaho). Two additional affected livestock herds occurred in the Idaho feed ground-HD in years after the feeding ground closed (Fig 1). No brucellosis positive livestock were detected between 1990 and 2001. Affected livestock herd sizes ranged from roughly 30 to 2300 and within herd brucellosis seroprevalence ranged from less than 0.01 to 0.22. Descriptions of elk-livestock comingling, testing history, calving history, and time spent in the DSA for each affected livestock herd’s initial *B*. *abortus*-positive animal helped to narrow identification of a spillover season for only 11 of the affected herds (Table B in S1 Appendix). Four of the affected livestock herds were probably infected in winter, because elk were being fed during this time in close proximity to the affected livestock or because elk were seen comingling with the affected livestock during winter. Two of the affected livestock herds were probably infected in the spring, because elk were seen on spring grazing property or in close proximity to the affected livestock during the spring months. One affected livestock herd was probably infected in the summer, because the initial affected animal was moved from outside the DSA to summer grazing inside the DSA only months before testing positive. Four of the affected livestock herds were probably affected sometime during spring or summer, based on descriptions of movement from outside the DSA to spring/summer grazing inside the DSA, successful calving in early spring (before testing positive in the late fall), or previous negative test history. The remaining 10 affected livestock herds lacked any information to identify a spillover season. We also found that all detection methods detected similar numbers of affected livestock herds (each method detected 3–5 affected herds; Table B in S1 Appendix).

Seroprevalence data in elk suggests elk exposure to *B*. *abortus* has increased over time in many HDs distant from the feeding grounds, including HDs east, west and north of Yellowstone National Park (Fig 2; also see S3 Fig for a summary of model uncertainty and S4 Fig for an example of the sampling effort). Even with these increases, estimates of seroprevalence in 2014 were still highest among fed elk: estimated median seroprevalence was 0.29 in feed ground HDs compared to 0.11 across all HDs without feeding grounds. Across all years, the median seropositive elk density was 0.14 elk/km^2^, but values ranged from roughly zero to 24 elk/km^2^, with hotspots around the National Elk Refuge, Wyoming state-operated feeding grounds, and Madison Valley in Montana (S5 Fig). In HDs with and without feeding grounds median spring SWE was 28 cm and 25 cm in years without affected livestock herds compared to 15 cm and 20 cm in years with affected herds, respectively. There was little difference in average May NDVI between HDs with and without feeding grounds or between years with and without affected livestock herds.

**Fig 3:**
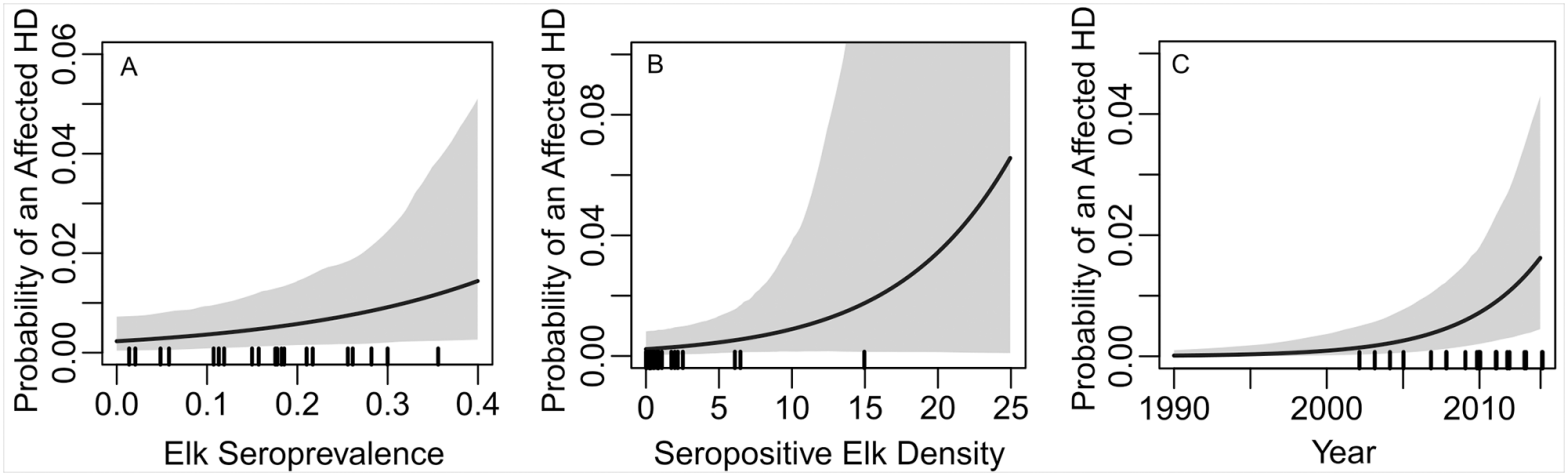
Estimated relationships with the probability of a brucellosis-affected hunt district (HD). (A) elk brucellosis seroprevalence, (B) seropositive elk density, and (C) year. Note y axis differ among panels. Grey shaded areas are 95% credible intervals. Probability of an Affected HD = probability of an HD having brucellosis-affected livestock detected. Rug plots depict the x axis values associated with affected HDs.

**Fig 4:**
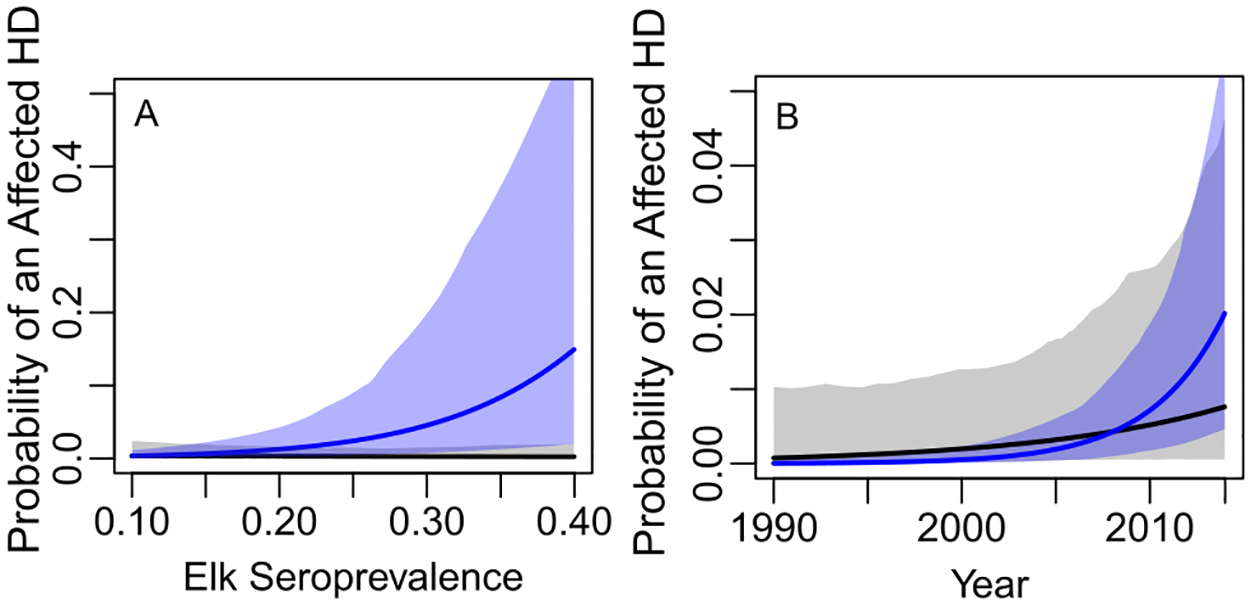
Interacting effects on the probability of a brucellosis-affected hunt district (HD). (A) elk brucellosis seroprevalence × fed, and (B) year × fed. Note y axes differ among panels. Black and blue lines (mean) and shaded areas (95% credible intervals) describe the estimated relationships for fed and unfed elk, respectively. Probability of an Affected HD = probability of an HD having brucellosis-affected livestock detected. Fed = elk feeding ground present.

As expected, elk seroprevalence, density of seropositive elk, and year were estimated to be positively related to occurrences of affected HDs (HD having brucellosis-affected livestock detected; see Table 1 for parameter estimates on log odds scale and Fig 3 for estimated relationships on the probability scale). Most of the affected livestock herds were detected in HD-years with less than 2.5 seropositive elk/km^2^ and none of the affected livestock herds were detected in HD-years with greater than 15 seropositive elk/km^2^. In Fig 3 the relationship extends beyond 15 seropositive elk/km^2^ to cover the full range of estimated seropositive elk densities (S5 Fig) and highlight model uncertainty at high densities (e.g., wide 95% credible intervals [CI]). The effect size for seroprevalence was small, but became more important after accounting for fed/unfed status (Table 1, Fig 4). This model suggests that in HDs without feeding grounds the odds of an affected HD increased roughly 14% (e^0.13^ = 1.14; 95% CI = 1.06, 1.23) with every 1% increase in elk seroprevalence, but in HDs with feeding grounds, occurrences of affected HDs were not related to elk seroprevalence. We also estimated a steeper rate of increase in affected HDs over time for HDs without feeding grounds compared to HDs with feeding grounds, though the 95% CI of the year by feed ground interaction just overlapped zero (Table 1, Fig 4). From this model, in HDs without feeding grounds the odds of an affected HD increased roughly 31% (e^0.27^ = 1.31; 95% CI = 1.15, 1.49) with every additional year (Table 1, Fig 4).

The relationship between spring SWE and occurrences of affected HDs was estimated to be more strongly negative than positive in HDs with feeding grounds (Table 1). In these areas, the odds of an affected HD could decrease by more than half (e^(-0.10–0.67)^ = 0.46; 95% CI = 0.09, 1.54) with every increase in 10 cm of spring SWE. While in HDs without feeding grounds, the odds of an affected HD was likely to decrease by only 10% (e^(-0.10)^ = 0.90; 95% CI = 0.68, 1.17) with every increase in 10 cm of spring SWE. The parameter estimate for NDVI, however, was not strongly negative or positive (Table 1).

Posterior predictive checks indicated good model fit (Bayesian p-values > 0.40; S1 Table) and our comparison of models with and without random effects revealed small differences in the estimated odds of an affected HD in most cases (less than 4.2% difference; S1 Table). The differences were slightly larger for models with seropositive elk density and the spring SWE by feed ground interaction (9.1% and 7.7%, respectively), but parameter uncertainty was high and 95% CI overlapped zero for these variables in both model scenarios (S1 Table). For all variables, estimated 95% CI were wider with random effects included in the models (Table 1 and S1 Table).

## Discussion

Occurrences of brucellosis-affected livestock herds within the DSA have been relatively rare, but on the rise since the early 2000’s. The vast majority of these affected livestock herds (17 of 21) occurred in elk hunt districts (HDs) without operating elk feeding grounds (Fig 1), where brucellosis seroprevalence in elk has been increasing (Fig 2). In these HDs, the mean probability of having an affected livestock herd was estimated to increase from roughly 0.01 to 0.08 with an increase in elk seroprevalence from 0.20 to 0.35 (Fig 4). These probabilities should be interpreted with caution, however, given there were only 21 occurrences of affected livestock herds and estimated parameter uncertainty was high. Focusing instead on the broad patterns, our results suggest that spillover from elk to livestock could occur more often in areas with increasing seroprevalence in unfed elk, but that occurrences of brucellosis-affected livestock are still likely to be rare overall. In contrast to unfed elk, fed elk seroprevalence was not important to occurrences of affected livestock herds (Fig 4), probably because fed elk have had consistently higher levels of exposure to *B*. *abortus* and there were only four affected livestock herds in feed ground HDs across the study period. Feeding grounds help to control elk distribution away from livestock and to reduce comingling. Additionally, there is little tolerance for elk on private lands in the feeding ground area, and WGFD personnel actively haze elk away from livestock. This elk-livestock separation during the feeding season (roughly January through early April [38]) could explain why fed elk HDs have had fewer affected livestock herds, despite also having higher levels of elk seroprevalence. However, feeding grounds have likely served as foci of brucellosis in the GYA, contributing to disease spread in elk and spillover to livestock in many of the unfed elk HDs (see [7] for evidence of brucellosis spread from fed to unfed elk). In this case, feeding grounds could incur costs across the broader region, in terms of disease impacts on elk and livestock, while also being protective to livestock at a local level.

Feeding grounds may have seeded and intermittently fueled brucellosis in elk across the region, but evidence suggests that transmission has also been occurring locally [7] and that transmission rates are positively related to elk density and group size [18–20]. Elk densities have been declining over time in several high profile elk populations around Yellowstone National Park, while other elk management units across the study area have experienced growing elk populations (e.g. [39]) and group sizes that are, in some areas, largely out of ‘management control’ because of limited hunting access [40]. In other areas, high elk densities have been the direct goal of management. In either case, high densities of elk could perpetuate elk to elk transmission of *B*. *abortus* in unfed elk and increase the risk of spillover to livestock. Our examination of seropositive elk density suggests a positive relationship with occurrences of affected livestock herds, but there was a high degree of parameter uncertainty (Table 1 and Fig 3). This uncertainty was expected given the rarity in occurrences of affected livestock herds and the possibility of elk movement between HDs during the latter portion of the transmission period. The degree of movement occurring across the GYA during this time is unknown, but likely to be highly variable among individuals, years, and HDs. Future work should examine how elk movement and connectivity across the region change throughout the transmission period to understand the areas and times that are most important for spatial spread of brucellosis in elk.

Although cases of affected livestock herds are thought to be a result of spillover transmission from elk, the effects of elk seroprevalence were still relatively weakly estimated. This may be due to fine scale factors that affect elk distributions and elk-livestock interaction such as human activity levels, fencing, location of shared water sources, mineral supplements, crops or haystacks, and local cattle densities. We did not address these questions because our data were limited to a coarser spatial resolution to identify broad patterns of livestock risk across the entire DSA. Future case-control studies could help elucidate the importance of these variables, while controlling for elk density, seroprevalence, and snow accumulation. These studies, however, would require information about both brucellosis-positive and negative livestock herds that is often not available for public dissemination (e.g., specific herd locations and disease testing schedules) and would require specific knowledge about where spillover occurred. Yet for many of the affected livestock herds, winter feeding and spring grazing occurred in multiple disparate locations and for roughly half of the affected herds it was not clear which was the probable location of infection (Table B in S1 Appendix). Additional detail regarding animal testing, timing of calving, grazing locations, and movement histories would be necessary to refine spillover location and timing. This level of detail could help us understand whether transmission occurs more often on public grazing allotments or private pastures, and whether spring is a riskier time for lower elevation allotments and summer is riskier for high elevation allotments. The start of livestock grazing on most national forest allotments around the elk feeding grounds occurs on June 15 to reduce brucellosis transmission risk. However, access to grazing begins earlier on other federal allotments in other areas of the GYA and livestock presence on private land occurs in some areas throughout the transmission period. A further understanding of spillover timing could help refine allotment-access dates, in an effort to both maximize grazing and reduce risk.

Our model of spring snow provided only weak support of a spring snow effect on livestock risk overall, but suggests that spring snow was more important to livestock brucellosis in feed ground HDs when SWE was less than 25 cm. Snow has been shown to affect ungulate mobility [41], but individual movement may increase during times of low snow as less energy is required to search for higher quality forage or to escape from predators. Recent evidence suggests that fed elk move significantly more and farther away from feeding grounds in times of low snow (Merkle et al. *in Review*), and wandering fed elk have been found on private property near livestock (WGFD *unpublished data*). In this case, warmer winters predicted with climate change could lead to more elk-livestock comingling and small increases in the risk of spillover to livestock around the feeding grounds. Though we did not find strong snow effects in unfed elk HDs, lower snow levels have been associated with larger unfed elk groups [23] and larger groups have been linked to higher levels of brucellosis seroprevalence [19,20]. If these conditions (larger elk groups and higher seroprevalence) occur on elk winter range near livestock properties, it could increase the risk of spillover to livestock, but further research is needed to link livestock outbreaks to large elk groups.

Overall, our study suggests that focusing brucellosis mitigation efforts on non-feed ground HDs with high elk seroprevalence and high elk densities could further reduce the rare probability of brucellosis spillover from elk to livestock. Focused mitigation of livestock risk in these HDs could include activities to reduce elk-livestock comingling (e.g., fence haystacks, hire herders, depredation elk hunts; see [42] for these and other potential mitigation activities) or delaying turnout of livestock on high risk grazing allotments (e.g., from spring to mid-June when transmission risk is lower). As risk to livestock is generally low, however, the cost of mitigation activities may not be justified for some livestock producers in low risk areas (see [42,43] for a cost-benefit analysis of brucellosis prevention activities). For direct reductions of elk seroprevalence or the density of seropositive individuals, economically feasible methods of disease mitigation are generally limited to harvest management, whereby wildlife agencies use hunters to reduce elk numbers and disperse large aggregations of elk. Though hunting access limitations have made targeting certain elk populations on private land challenging, wildlife managers have been working to incentivize hunting on private lands with hunt coordinators who work on opening access for hunters where elk aggregate. Monitoring elk distributions and brucellosis seroprevalence in these cases will be important to determine the success of these methods, and adaptive disease management will be necessary to maximize mitigation efficiency across the changing landscape of risk.

## Supporting information

S1 Appendix Livestock brucellosis detection methods and summary of affected livestock herd epidemiological reports. (DOCX)

S2 Appendix Diagnostic tests for seropositive elk by state and year. (XLSX)

S3 Appendix Examining associations between wolf abundance and occurrences of affected livestock herds. (DOCX)

S1 Fig County level total cattle and cow only densities. Using the U.S. Department of Agriculture’s National Agricultural Statistics Service data on total cattle (including calves) and total cows only, retrieved from https://quickstats.nass.usgs.gov, we calculated (A) total cattle density by county and (B) total cow only density by county for all counties that overlap with the DSA. DSA = designated surveillance area for brucellosis in livestock. YNP = Yellowstone National Park. GTNP = Grand Teton National Park (some cattle grazing occurs in GTNP). The elk feed ground in Idaho is no longer operational.
(TIF)

S2 Fig Comparison of diagnostic tests and example effects of increased overall sensitivity and specificity on pattern of increasing seroprevalence in elk. Both panels depict elk seroprevalence over time for one Wyoming hunt district (HD). Blue dots in both panels represent seroprevalence determined from a combination of diagnostic tests as outlined in S2 Appendix (these data represent Wyoming’s final determination of seropositivity), and grey lines represent the fitted relationships of these data over time. Panel (A) compares Wyoming’s final determination of seropositivity to the seroprevalence obtained from the card test only (yellow) and rivanol test only (turquoise). Note there were no card tests run during 2012–2014 and no rivanol tests run during 2012 or 2014. Dot alignment with time was adjusted slightly to aid in the comparisons. Fitted relationships (lines) were obtained from a logistic regression of seroprevalence over time. Panel (B) compares Wyoming’s final determination of seropositivity to seroprevalence adjusted for sensitivity (Se) and specificity (Sp) using (Seroprevalence_WYfinal_ + Sp– 1) / (Se + Sp– 1). Light blue dots = seroprevalence adjusted for Se of 0.95 and Sp of 0.90 across all years. Temporal pattern in increasing seroprevalence is still apparent after this adjustment. Orange dots = seroprevalence adjusted for Se of 0.975 and Sp of 0.925 for years 2006 through 2014. Vertical dotted line at year 2005 indicates hypothetical time when the accuracy of diagnostic tests improved. Blue lines = binomial 95% confidence interval. Grey shaded region = 95% credible intervals from Bayesian model.
(TIF)

S3 Fig Elk seroprevalence model uncertainty per hunt district (HD). Uncertainty was averaged across the study period (1990–2014) for each HD. Std. Dev. = Standard deviation. YNP = Yellowstone National Park. GTNP = Grand Teton National Park. The elk feed ground in Idaho is no longer operational.
(TIF)

S4 Fig Modeled and empirical seroprevalence data (and sample sizes) in elk for four hunt districts (HDs) without elk feeding grounds. HDs shown are the same identified in Fig 2 of main text. Blue dots = empirical data. Blue lines = binomial 95% confidence interval. Sample sizes are shown above confidence intervals. Grey dots = modeled estimates of seroprevalence. Grey shaded region = 95% credible intervals.
(TIF)

S5 Fig Brucellosis seropositive elk density (elk/km^2^) per hunt district (HD). Estimates were averaged across the study period (1990–2014) for each HD. The highest average seropositive elk density (20.6 elk/km^2^) occurred on the National Elk Refuge just south of GTNP. YNP = Yellowstone National Park. GTNP = Grand Teton National Park. The elk feed ground in Idaho is no longer operational.
(TIF)

S1 Table Posterior predictive checks, tests of model support and parameter comparison to models without random effects. (DOCX)
