# Supplementary material for: Shifting brucellosis risk in livestock coincides with spreading seroprevalence in elk
Source: PLoS One. 2017 Jun 13;12(6):e0178780. doi: 10.1371/journal.pone.0178780 (PMC5469469; doi:10.1371/journal.pone.0178780)
Supplement: S1 Appendix — (DOCX) [file pone.0178780.s001.docx]

**S1 Appendix: Livestock brucellosis detection methods and summary of affected livestock herd epidemiological reports.**

**Table A. Descriptions of detection methods used to identify infected livestock.**

| **Method of Detection** | **Description** |
| --- | --- |
| FPT market | First point of concentration testing at markets where there is a convergence of animals from many locations. |
| MCI | Market cattle identification initiated at slaughter/auction facilities for cattle identified as coming from within the designated surveillance area (DSA). |
| Epidemiological trace out | Trace out testing for herds containing animals sourced from affected herd. |
| Epidemiological trace in | Trace in testing for herds that were a source of animals to the affected herd. |
| Herd plan | Testing is outlined in herd management plans because of proximity to exposed elk. |
| Movement out of DSA | Testing within 30 days after moving livestock from DSA to an outside location. |
| Change of ownership | Testing within 30 days after DSA livestock are sold to a new owner. |
| Note: The specific timing and test eligible (animal sex, age) requirements or guidelines for these methods may differ among states. | |

**Table B. Summary of epidemiological reports on brucellosis-affected livestock herds in the Designated Surveillance Area (DSA).**

| **State** | **Year of  Detection** | **Method of Detection** | **Season of Infection (SI)** | **Explanation for SI** | **Herd Type** | **Sero+ (%)** | **Herd Size** | **Difference in Sero+ reported in Rhyan et al. 2013*** |
| --- | --- | --- | --- | --- | --- | --- | --- | --- |
| ID | 2002 | Herd plan | **W** | Elk were fed nearby during winter. Affected animal aborted in December. Previous disease tests were negative. | Cattle | 12.0 | 50 | Same as Rhyan et al. |
| WY | 2003 | FPT market | Sp/Su/W | No information. | Cattle | 8.7 | 391 | Rhyan et al. included trace out herd below (greyed row) |
| WY | 2004 | Epi trace out | Not a separate outbreak. | Linked to above herd. | Cattle | 50.0 | 12 |  |
| WY | 2004 | Movement out of DSA | Sp/Su/W | No information. | Cattle | 0.9 | 105 | Rhyan et al. included trace out herd below (greyed row) |
| WY | 2004 | Epi trace out | Not a separate outbreak. | Linked to above herd. | Cattle | 1.1 | 370 |  |
| ID | 2005 | MCI | **W** | Temporary winter supplemental feed ground for elk was nearby; elk were seen in livestock feeding areas. | Cattle | 22.0 | 41 | Rhyan et al. included trace out herd below (greyed row) |
| ID | 2005 | Epi trace out | Not a separate outbreak. | Linked to above herd. | Cattle | 12.0 | 8 |  |
| MT | 2007 | Change of ownership | Sp/Su/W | Infection probably occurred in 2005 (reactor cow aborted late 2005 and 2006); season of infection unknown. | Cattle | 1.2 | 600 | We included additional sero+ animals that were detected in subsequent tests |
| MT | 2008 | Herd plan | Sp/Su/W | No information | Cattle | 2.9 | 34 | Same as Rhyan et al. |
| WY | 2008 | FPT market | Sp/Su/W | Infection probably occurred in 2007 (reactor cow aborted late 2007); season of infection unknown. | Cattle | 5.5 | 656 | Same as Rhyan et al. |
| ID | 2009 | MCI | **Su** | Affected animal moved from outside DSA to summer grazing in DSA near elk with higher seroprevalence. No known winter comingling of elk and cattle. | Cattle | 1.4 | 590 | Same as Rhyan et al. |
| MT | 2010 | Herd plan | Sp/Su/W | No information. | Bison | 0.3 | 967 | Rhyan et al. summed both 2010 bison herds from MT, and reported year as 2011. But cases were detected in 2010 and determined to be separate. |
| MT | 2010 | Epi trace out | Sp/Su/W | No information. | Bison | 0.2 | 452 | See above |
| WY | 2010 | FPT market | Sp/Su/W | Elk roam year round in this area; no specific comingling event witnessed. | Cattle | 1.1 | 450 | Same as Rhyan et al. |
| WY | 2010 | Change of ownership | Sp/Su/W | Elk roam year round in this area; no specific comingling event witnessed. | Bison | 11.5 | 1204 | Same as Rhyan et al. |
| WY | 2011 | FPT market | Sp/Su/W | Elk roam year round in this area; no specific comingling event witnessed. | Cattle | 1.1 | 567 | Similar to Rhyan et al. (% Sero+ was reported as 0.9) |
| WY | 2011 | Change of ownership | **Sp** | Elk and cattle seen comingling spring of 2011. | Cattle | 1.1 | 280 | Similar to Rhyan et al. (% Sero+ was reported as 1.2) |
| MT | 2011 | Change of ownership | **Late W** | Elk and cattle shared water source in late winter. | Cattle | 2.9 | 204 | Similar to Rhyan et al. (% Sero+ was reported as 2.0) |
| ID | 2012 | Herd plan | **W** | Herd testing initiated in 2010. Elk were seen comingling with livestock during winter of 2011. | Bison | 0.7 | 267 | Same as Rhyan et al. |
| ID | 2012 | MCI | **Sp** | Sporadic elk were seen near spring grazing location, which was adjacent to known elk winter range. | Cattle | 9.2 | 65 | Rhyan et al. included calves in herd size (% Sero+ was reported as 5.8) |
| MT | 2013 | Change of ownership | **Late Sp/Su** | Affected animal successfully calved in March 2013; probably infected spring/summer 2013. | Cattle | 0.3 | 1114 | Not included in Rhyan et al. |
| MT | 2013 | Brucellosis free cert./Herd plan | **Sp/Su** | Likely infected during spring/summer of 2013 due to negative testing history. | Cattle | 0.2 | 557 | Not included in Rhyan et al. |
| MT | 2014 | Movement out of DSA | **Late Sp/Su** | Affected animal had previous negative test results in fall 2013, successfully calved spring 2014, and grazed in DSA late spring/summer 2014. | Cattle | 0.4 | 262 | Not included in Rhyan et al. |
| MT | 2014 | Movement out of DSA | **Sp/Su** | Spring/summer grazing occurred in DSA and elk seen in spring turn-out area. | Cattle | 0.04 | 2338 | Not included in Rhyan et al. |
| Notes: Sp/Su/W = Spring/Summer/Winter. Sp/Su/W indicates that epidemiological reports lacked detail necessary to attribute initial infection to a particular season. Shaded rows are animals that were not examined as a separate affected herd, because they were epidemiologically linked to a previously identified affected herd (in the row above). Detection methods described in Table A. SI = Season of infection. Epi = Epidemiological.  *Rhyan, JC, P Nol, C Quance, A Gertonson, J Belfrage, L Harris, K Straka, and S Robbe-Austerman. 2013. Transmission of brucellosis from elk to cattle and bison, Greater Yellowstone Area, USA, 2002–2012. Emerging Infectious Diseases, 19:1992–1995. | | | | | | | | |
