# Supplementary material for: Shifting brucellosis risk in livestock coincides with spreading seroprevalence in elk
Source: PLoS One. 2017 Jun 13;12(6):e0178780. doi: 10.1371/journal.pone.0178780 (PMC5469469; doi:10.1371/journal.pone.0178780)
Supplement: S3 Appendix — (DOCX) [file pone.0178780.s003.docx]

**S3 Appendix: Examining associations between wolf abundance and occurrences of brucellosis-affected livestock herds.**

We used December wolf counts, representing minimum pack sizes, from U.S. Fish and Wildlife Service (USFWS) reports to understand relationships between wolves and occurrences of brucellosis-affected livestock herds [1]. We used wolf pack home ranges (minimum convex polygons) from these reports to estimate the proportion of each pack that occurred in our sampling units (elk hunt districts [HD]), and summed across packs to obtain wolf abundance by HD. Because minimum convex polygons do not describe variation in use within home ranges, we used this measure of wolf abundance as an index to describe broad spatial differences in wolf numbers. We then examined the interacting effects of elk density and wolf abundance on occurrences of brucellosis-affected livestock herds (Wolf × Elk Density) and the effects of wolf abundance while controlling for elk density (Wolf + Elk Density). See the main document for a description of the general livestock model framework and model of elk density. This model excluded the elk seroprevalence model because we were interested in the interaction with (or accounting for) elk density, not seropositive elk density.

**Table 1. Parameter estimates (log odds) for wolf-models of brucellosis-affected hunt districts (HD).**

| Model | Density Effect:  Posterior mean  (95% CI) | Wolf Effect:  Posterior mean  (95% CI) | Interaction:  Posterior mean  (95% CI) |
| --- | --- | --- | --- |
| Elk Density × Wolf + RE | 0.01  (-0.07, 0.08) | 0.10  (-0.06, 0.27) | -0.006  (-0.02, 0.006) |
| Elk Density + Wolf + RE | 0.0008  (-0.06, 0.05) | 0.06  (-0.04, 0.14) | - |
| Notes: Main effects describe the log odds of a brucellosis affected herd district per 1 unit increase in the explanatory variable. The interaction describes the change in the main effect for elk density with increasing wolf abundance. RE = Random effect. N = 2050. | | | |

References

1. U.S. Fish and Wildlife, Montana Fish Wildlife & Parks, Nez Perce, National Park Service, Blackfeet Nation, Confederated Salish and Kootenai Tribes, et al. Rocky mountain wolf recovery 2014 interagency annual report. Helena, MT; 2015.
