# Supplementary figures and images for: Shifting brucellosis risk in livestock coincides with spreading seroprevalence in elk
Source: PLoS One. 2017 Jun 13;12(6):e0178780. doi: 10.1371/journal.pone.0178780 (PMC5469469; doi:10.1371/journal.pone.0178780)

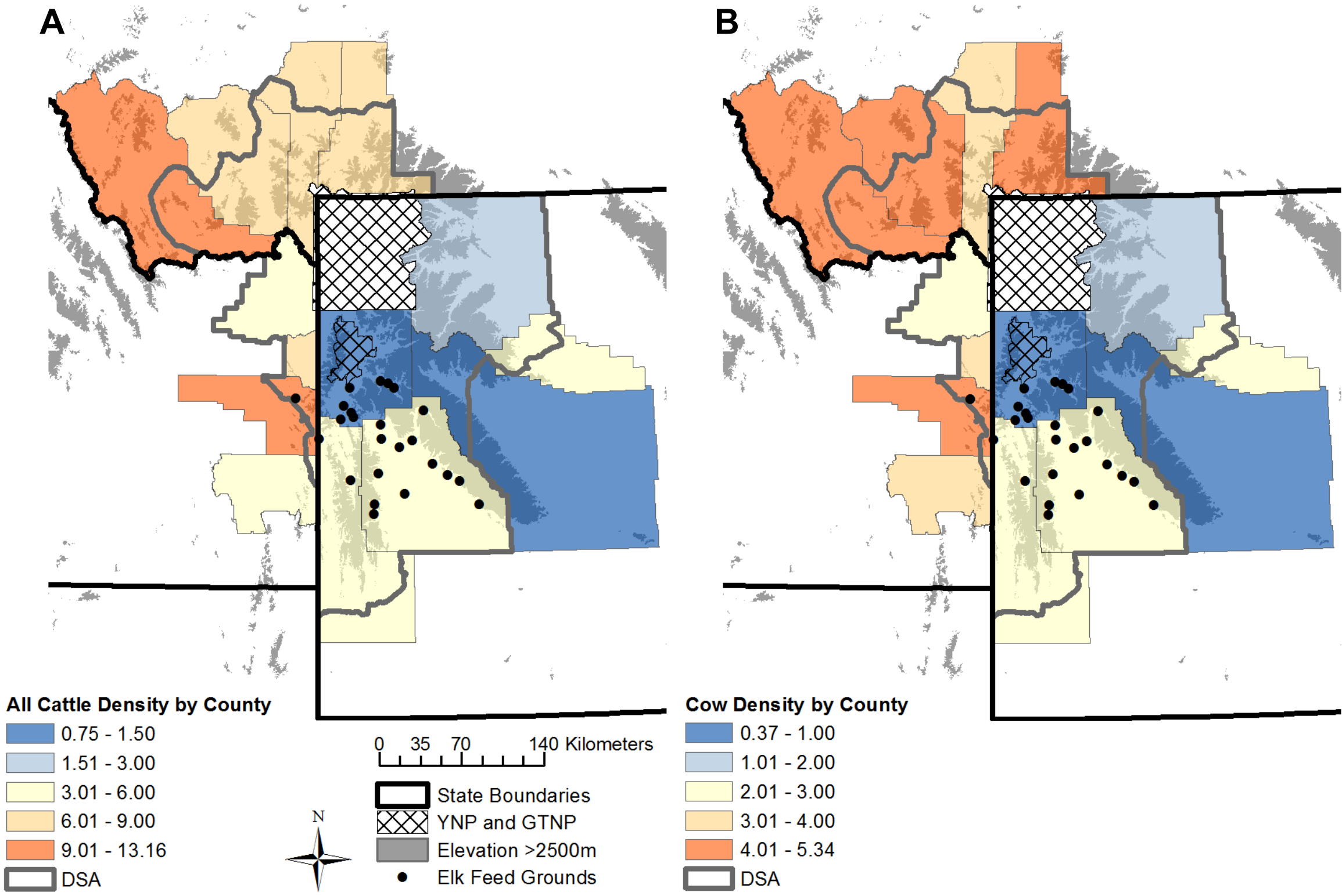

Supplement: S1 Fig — Using the U.S. Department of Agriculture’s National Agricultural Statistics Service data on total cattle (including calves) and total cows only, retrieved from https://quickstats.nass.usgs.gov, we calculated (A) total cattle density by county and (B) total cow only density by county for all counties that overlap with the DSA. DSA = designated surveillance area for brucellosis in livestock. YNP = Yellowstone National Park. GTNP = Grand Teton National Park (some cattle grazing occurs in GTNP). The elk feed ground in Idaho is no longer operational. (TIF) [file pone.0178780.s004.tif]

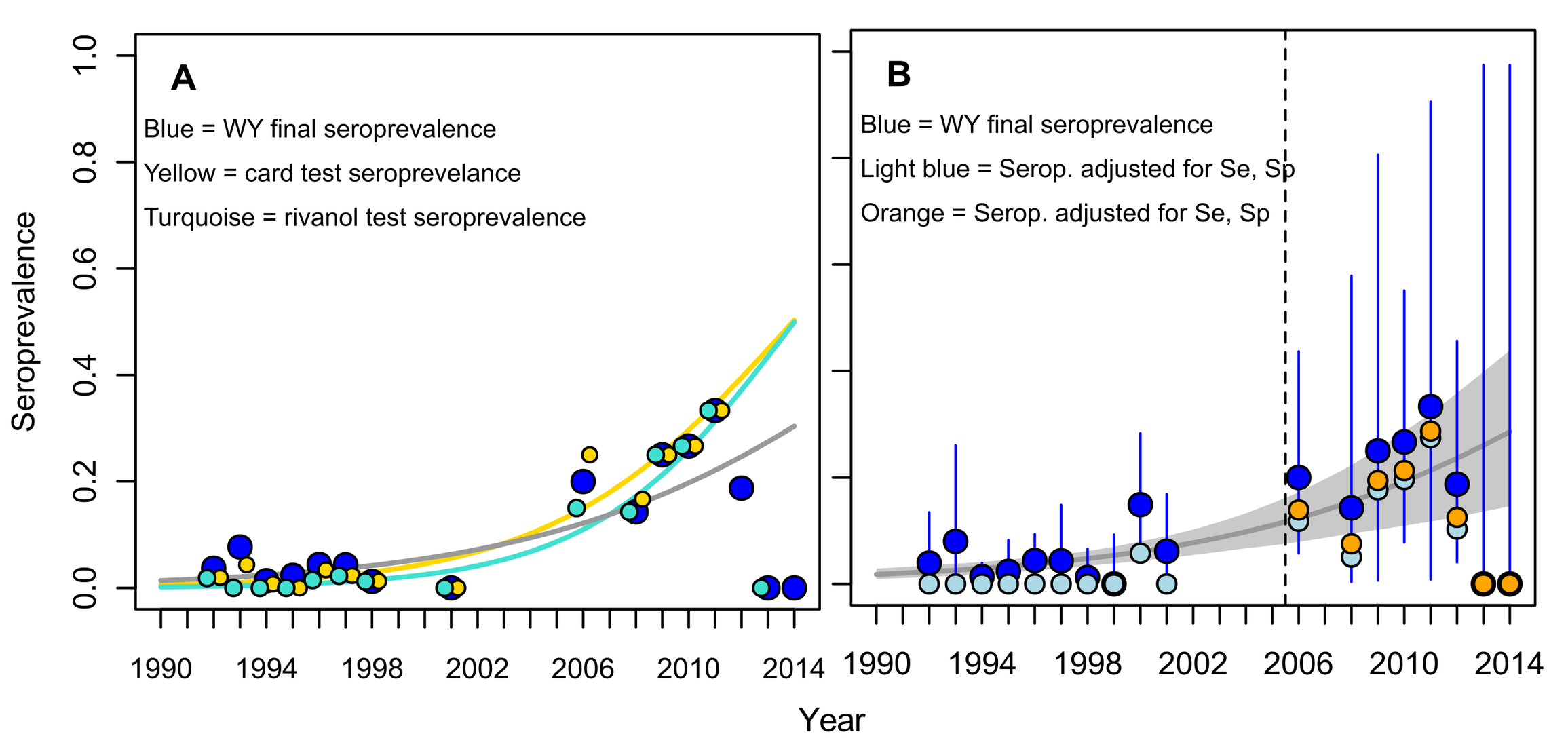

Supplement: S2 Fig — Both panels depict elk seroprevalence over time for one Wyoming hunt district (HD). Blue dots in both panels represent seroprevalence determined from a combination of diagnostic tests as outlined in S2 Appendix (these data represent Wyoming’s final determination of seropositivity), and grey lines represent the fitted relationships of these data over time. Panel (A) compares Wyoming’s final determination of seropositivity to the seroprevalence obtained from the card test only (yellow) and rivanol test only (turquoise). Note there were no card tests run during 2012–2014 and no rivanol tests run during 2012 or 2014. Dot alignment with time was adjusted slightly to aid in the comparisons. Fitted relationships (lines) were obtained from a logistic regression of seroprevalence over time. Panel (B) compares Wyoming’s final determination of seropositivity to seroprevalence adjusted for sensitivity (Se) and specificity (Sp) using (SeroprevalenceWYfinal + Sp– 1) / (Se + Sp– 1). Light blue dots = seroprevalence adjusted for Se of 0.95 and Sp of 0.90 across all years. Temporal pattern in increasing seroprevalence is still apparent after this adjustment. Orange dots = seroprevalence adjusted for Se of 0.975 and Sp of 0.925 for years 2006 through 2014. Vertical dotted line at year 2005 indicates hypothetical time when the accuracy of diagnostic tests improved. Blue lines = binomial 95% confidence interval. Grey shaded region = 95% credible intervals from Bayesian model. (TIF) [file pone.0178780.s005.tif]

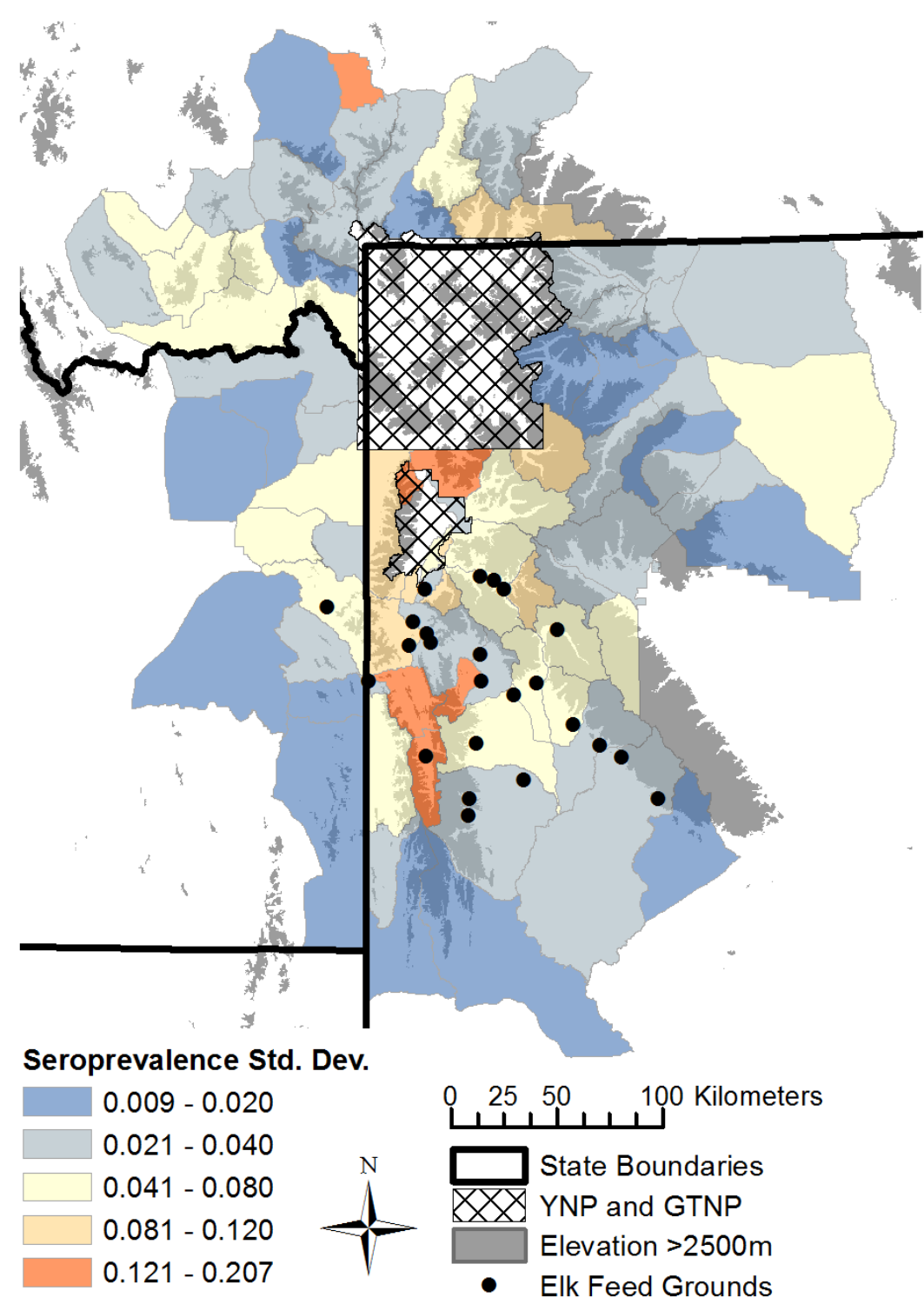

Supplement: S3 Fig — Uncertainty was averaged across the study period (1990–2014) for each HD. Std. Dev. = Standard deviation. YNP = Yellowstone National Park. GTNP = Grand Teton National Park. The elk feed ground in Idaho is no longer operational. (TIF) [file pone.0178780.s006.tif]

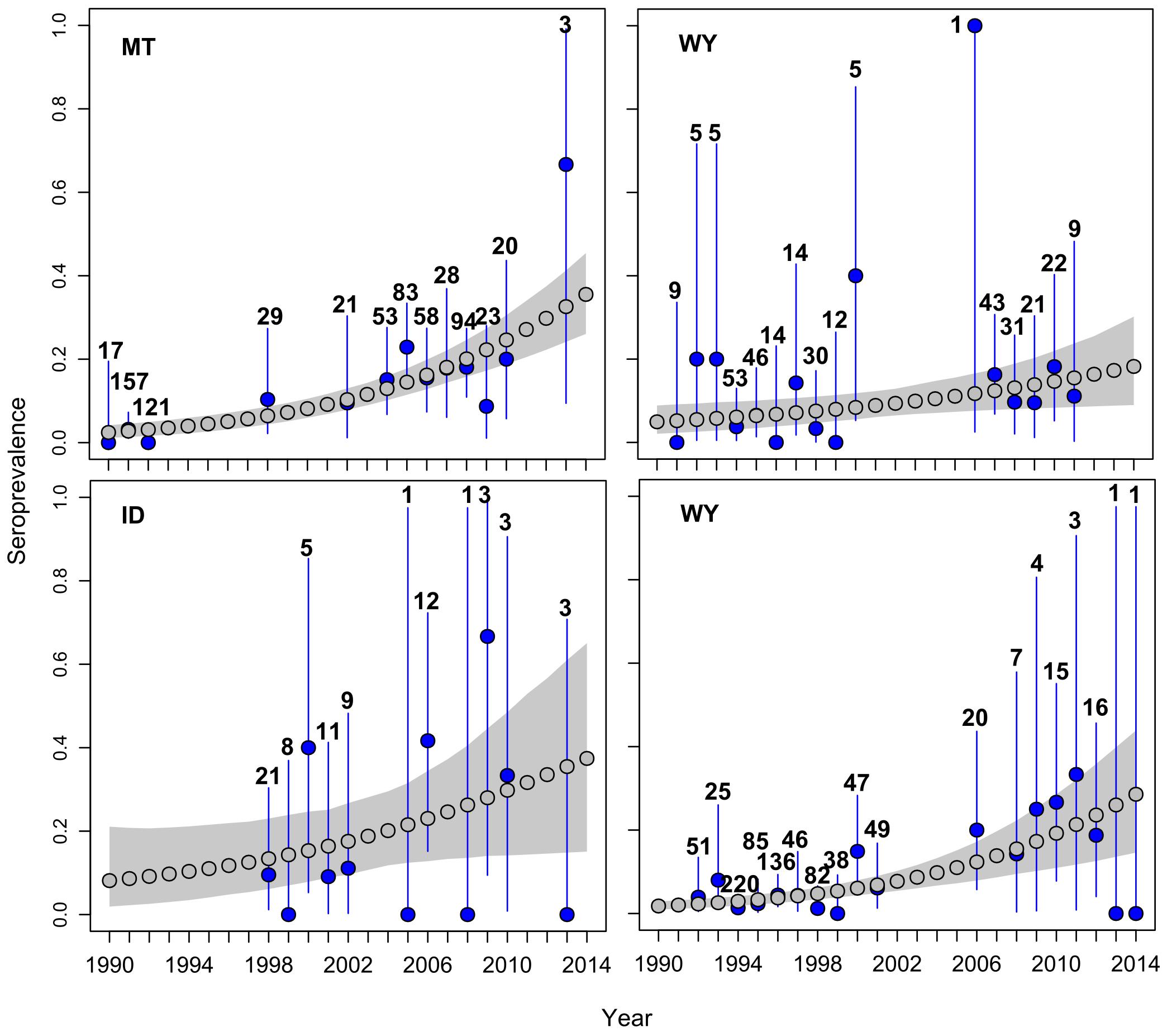

Supplement: S4 Fig — HDs shown are the same identified in Fig 2 of main text. Blue dots = empirical data. Blue lines = binomial 95% confidence interval. Sample sizes are shown above confidence intervals. Grey dots = modeled estimates of seroprevalence. Grey shaded region = 95% credible intervals. (TIF) [file pone.0178780.s007.tif]

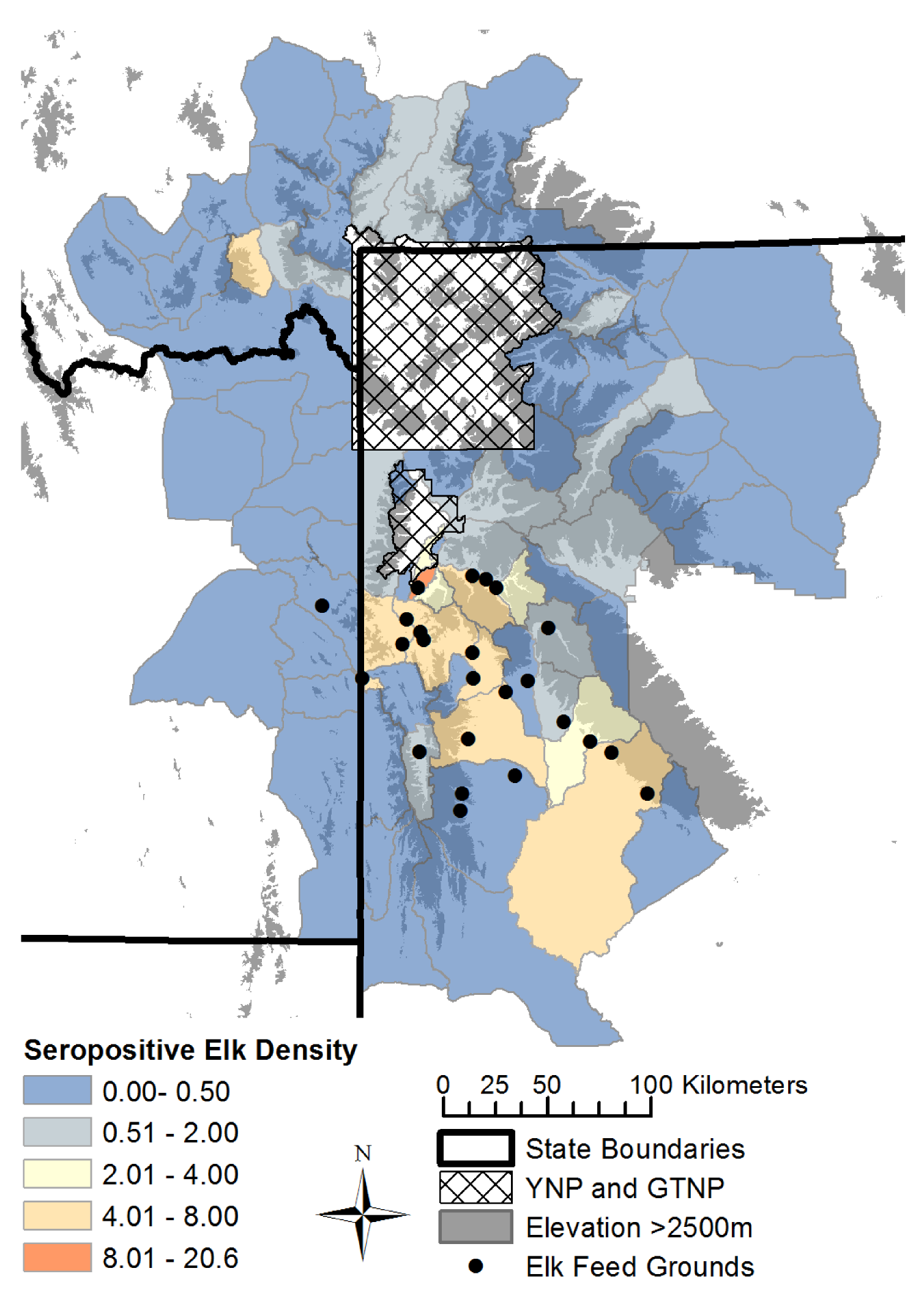

Supplement: S5 Fig — Estimates were averaged across the study period (1990–2014) for each HD. The highest average seropositive elk density (20.6 elk/km2) occurred on the National Elk Refuge just south of GTNP. YNP = Yellowstone National Park. GTNP = Grand Teton National Park. The elk feed ground in Idaho is no longer operational. (TIF) [file pone.0178780.s008.tif]
