# Supplementary material for: Shifting brucellosis risk in livestock coincides with spreading seroprevalence in elk
Source: PLoS One. 2017 Jun 13;12(6):e0178780. doi: 10.1371/journal.pone.0178780 (PMC5469469; doi:10.1371/journal.pone.0178780)
Supplement: S1 Table — (DOCX) [file pone.0178780.s009.docx]

**S1 Table. Posterior predictive checks, tests of model support and parameter comparison to models without random effects.**

| Model | Δ DIC  (DIC_N_ – DIC_F_) | Bayesian p-value | Main Effect W/O RE: Posterior mean (95% CI) | % Difference (in odds) to Main Effect W/ RE | Interaction W/O RE: Posterior mean (95% CI) | % Difference (in odds) to Main Effect W/ RE |
| --- | --- | --- | --- | --- | --- | --- |
| Elk Seroprevalence + RE | 14.8 | 0.45 | 0.05  (0.01, 0.08) | 0.1 | - | - |
| Seropositive Elk Density + RE | 19.8 | 0.45 | 0.04  (-0.09, 0.13) | 9.1 | - | - |
| Year + RE | 38.6 | 0.48 | 0.19  (0.01, 0.30) | 1.0 | - | - |
| NDVI+ RE | 13.8 | 0.46 | 0.006  (-0.003, 0.004) | 1.4 | - | - |
| Elk Seroprevalence × Fed + RE | 30.4 | 0.40 | 0.12  (0.07, 0.18) | 1.0 | -0.11  (-0.21, -0.02) | 4.1 |
| Year × Fed + RE | 38.2 | 0.42 | 0.26  (0.14, 0.39) | 1.0 | -0.19  (-0.40, 0.02) | 2.0 |
| Sp. SWE × Fed + RE | 12.3 | 0.41 | -0.11  (-0.35, 0.09) | 1.0 | -0.75  (-2.0, 0.12) | 7.7 |
| Notes: DIC_N_ = null model DIC (Deviance Information Criterion). DIC_F_ = full model DIC. Null models included only an intercept term in the affected livestock herd level. RE = random effect. W/O RE = from model without random effect on sampling unit. W/ RE = from model with random effect on sampling unit. % Difference (in odds) was calculated as the absolute value of 100 – (100*(exp(main effect w/RE) / exp(main effect w/o RE))), where exp (or exponential) was used to obtain values on the odds scale. Fed = elk feeding ground present. NDVI = Normalized Difference Vegetation Index. Sp. SWE = average spring snow water equivalent. Bayesian p-values near 0.5 indicate good model fit. | | | | | | |
